# The AP-1 repressor protein, JDP2, potentiates hepatocellular carcinoma in mice

**DOI:** 10.1186/1476-4598-9-54

**Published:** 2010-03-09

**Authors:** Keren Bitton-Worms, Eli Pikarsky, Ami Aronheim

**Affiliations:** 1 Department of Molecular Genetics, The Rappaport Family Institute for Research in the Medical Sciences, Technion-Israel Institute of Technology, 1 Efron St Bat-Galim, Haifa 31096, Israel; 2 Department of Pathology and the Lautenberg Center for Immunology, IMRIC, Hebrew University/Hadassah Medical School, Jerusalem 91120, Israel

## Abstract

**Background:**

The AP-1 transcription factor plays a major role in cell proliferation, apoptosis, differentiation and developmental processes. AP-1 proteins are primarily considered to be oncogenic. Gene disruption studies placed c-Jun as an oncogene at the early stage of a mouse model of hepatocellular carcinoma. Mice lacking c-Jun display reduced number and size of hepatic tumors attributed to elevated p53 expression and increased apoptosis. This suggests that c-Jun inhibition may serve as a therapeutic target for liver cancer. The c-Jun dimerization protein 2, JDP2 is an AP-1 repressor protein that potently inhibits AP-1 transcription. On the other hand, the JDP2 locus was found at a recurring viral integration site in T-cell lymphoma. We sought to examine the potential of JDP2 to inhibit c-Jun/AP-1 oncogenic activity in mice. Towards this end, we generated a tetracycline inducible transgenic mouse expressing JDP2 specifically in the liver. We used diethylnitrosamine (DEN) injection to initiate liver cancer in mice and assessed the extent of liver cancer in JDP2-transgenic and wild type control mice by biochemical and molecular biology techniques.

**Results:**

JDP2-transgenic mice display normal liver function. JDP2-transgenic mice displayed potentiation of liver cancer, higher mortality and increased number and size of tumors. The expression of JDP2 at the promotion stage was found to be the most critical for enhancing liver cancer severity.

**Conclusions:**

This study suggests that JDP2 expression may play a critical role in liver cancer development by potentiating the compensatory proliferative response and increased inflammation in the DEN liver cancer model.

## Background

Hepatocellular carcinoma (HCC) is the fifth most frequent cancer. Approximately 500,000 cases of HCC are diagnosed each year and it is the third leading cause of cancer-related deaths [1]. The majority of cases of HCC are due to hepatitis B virus, hepatitis C infections and alcohol consumption [2]. During the course of chronic infection, continuous intra-hepatic inflammation maintains a cycle of liver cell destruction and regeneration that often terminates in HCC [3,4]. Various mouse models for HCC were developed to study the molecular mechanisms involved in various stages of liver cancer [5]. These mouse models mimic the etiology of liver cancer in man. Certain chemical carcinogens which induce hepatocyte DNA damage can result in HCC. Mice injected with a single injection of tumor initiator diethylnitrosamine (DEN) results in liver cancer [6-8]. Inflammation is a known factor that plays a casual role in cancer in general and liver cancer in particular [8-10].

AP-1 is one of the transcription factors found at the receiving end of multiple signaling pathways. AP-1 is composed of either homo or hetero dimers of basic leucine zipper (bZIP) family members [11]. Dimerization is required for specific binding to a DNA sequence known as the TPA response element (TRE) found in the promoter region of many genes. Although the role of AP-1 in cell proliferation is well established [12], activating mutations of this complex have never been found in human cancer. Most of the important insights regarding the specific function of AP-1 proteins have been established through the use of mice with either loss- or gain-of-function manipulations of various members of the bZIP protein family [13].

The most-studied member of the AP-1 family is c-Jun. Despite its ability to transform chicken embryo fibroblasts, c-Jun over-expression alone does not result in transformation of rodent fibroblasts. On the other hand, c-Jun cooperates with Ha-Ras in cell transformation [14]. In mice, c-Jun disruption causes embryonic lethality at midgestation. The affected embryos exhibit heart defects and arrested liver development [15]. Conditional c-Jun knockout in the liver of adult mice prevents the appearance of HCC in the DEN liver cancer model, implicating c-Jun as a crucial player in the initiation steps of liver carcinogenesis [16].

The c-Jun dimerization protein 2, JDP2, encodes an 18 kDa bZIP protein that interacts with c-Jun [17]. JDP2 can bind TRE DNA elements as well as the cyclic AMP response element (CRE) as a homo- or hetero dimer [17,18]. Upon dimerization with c-Jun, DNA binding is potentiated, but the transcription is inhibited [17]. JDP2 inhibits transcription by multiple mechanisms [17,19]. JDP2 is involved in cell differentiation processes, such as differentiation of skeletal muscle cells [20], osteoclasts [21] and in stress response to ultraviolet irradiation [22]. Various studies suggest that JDP2 has a dual role in malignant transformation. On the one hand, it is well established that JDP2 counteracts AP-1 transcription [17], and thus may interfere with the oncogenic properties of c-Jun. JDP2 has been found to inhibit cell transformation induced by Ras *in vitro *and in xenografts injected into SCID mice [23]. On the other hand, JDP2 has been identified as a candidate oncogene in a high-throughput screen based on viral insertional mutagenesis in mice [24-26]. Serial analysis of gene expression identified increased levels of JDP2 in a number of cancers including prostate, kidney, liver and skeletal muscle http://www.genecards.org/cgi-bin/carddisp.pl?gene=JDP2&search=JDP2. In order to examine whether JDP2 increased expression may be a coincidence or may play a role in liver cancer development, we have generated transgenic mice with liver specific JDP2 expression and examined the consequence in a chemically induced liver cancer model. We found that JDP2 increases liver cancer severity and that JDP2 expression at the promotion stage is most important for this activity.

## Results

**Figure 1:**
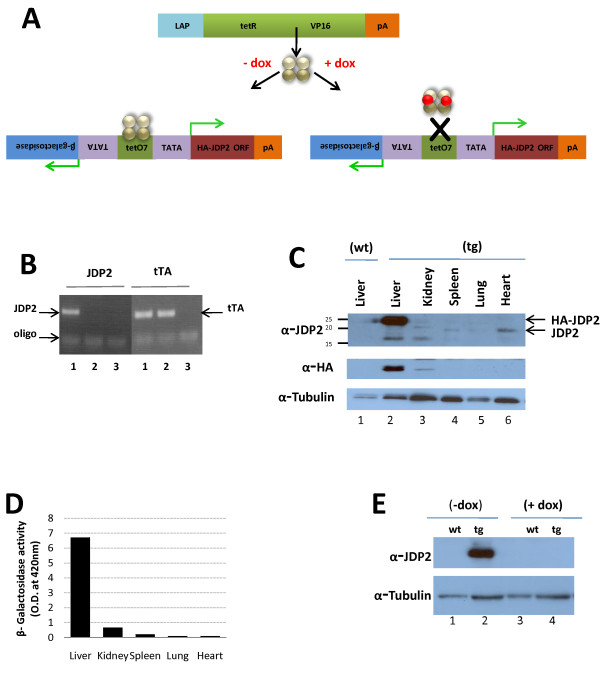
**Liver specific expression of JDP2 in JDP2-transgenic and control mice**. **A**. HA-JDP2 expressing mice responder line was generated in the pBIG bi-cistronic expression plasmid under the regulation of tetracycline response elements. The promoter co-regulates the expression of the β-galactosidase reporter together with HA-JDP2 protein. tTA-responder mice express the tetracycline activator (tTA) under the control of Liver Activating Protein promoter (LAP). tTA is expressed specifically in hepatocytes. The association of the tTA with the DNA is prevented in the presence of doxycycline thus results in shut-off transgene expression. **B**. Mouse tail genotyping by PCR was performed with specific oligonucleotides corresponding to the tTA driver (tTA, right panel) and JDP2-transgene (JDP2, left panel). DNA derived from either double transgenic mouse, JDP2-transgenic (lanes 1) or control wild type mouse (lanes 2) was used. Lanes 3 no DNA was added to the PCR mix. **C**. Western blot analysis with nuclear extract derived from the indicated tissues of JDP2-transgenic mouse (tg) and liver lysate from wild type mouse (wt). Membranes were probed with anti-JDP2 antibody (top panel) anti-HA antibody (middle panel) and anti-α-tubulin antibody (bottom panel) was used as loading control. The migration of HA-JDP2 transgene and endogenous JDP2 protein is indicated by an arrow at the right side of the top panel. **D**. β-galactosidase activity with cell lysate derived from the indicated tissues of JDP2-transgenic mouse. The O.D. value at 420 nm is shown. **E**. Western blot analysis with liver nuclear cell lysate derived from either wild type (wt) or JDP2-transgenic (tg) mice either untreated (-dox) or treated with doxycycline (+dox, 0.2 mg/ml) for 3 days. Membrane was probed sequentially with anti-JDP2 (top panel) followed by anti-α-tubulin (bottom panel) as loading control.

To establish the role of JDP2 over-expression in hepatocellular carcinoma, we have generated a tetracycline regulated JDP2 responder transgenic mouse line [27]. HA-JDP2 and the β-Galactosidase reporter are co-expressed under the control of the tetracycline regulator (Figure 1A). A mouse line showing germ line transmission was crossed with a LAP-driver mouse line expressing the tetracycline transactivator (tTA) in the liver [28]. Mice crosses were performed between matched gender homozygous LAP-driver mice and JDP2-heterozygous transgenic mice. Mice positive for both JDP2 transgene and tTA (Figure 1B) are expected to express HA-JDP2 in hepatocytes and are further designated JDP2-transgenic. The mating resulted the expected 50% Mendelian distribution of JDP2-transgenic (tg) and control tTA transgene (wt). Since male mice are more susceptible to liver cancer in the DEN model [29], all experiments described were performed with male mice. To study the HA-JDP2 specific expression in the liver, JDP2-transgenic mice were sacrificed and lysates derived from different tissues were separated by SDS-PAGE followed by Western blotting with either anti-HA or anti-JDP2 antibodies (Figure 1C). Whereas a strong cross reactive band at the expected 24 kDa size was observed with lysate derived from liver of JDP2-transgenic, no expression was observed in the liver of wild type mice. In addition, lysate derived from heart, lung and spleen of JDP2-transgenic mice did not display expression of the transgene however, endogenous JDP2 expression is readily observed at the expected 18 kDa size. Lysate derived from kidney, displayed a very low level of expression of the HA-JDP2 transgene (Figure 1C, anti-HA). Consistently, β-galactosidase activity of cell lysates derived from different tissues of JDP2-transgenic mice displayed high activity in the liver and at least 10 fold lower β-galactosidase activity was measured in the kidney and negligible activity was observed in heart, lung and spleen (Figure 1D). The LAP-tet driver mouse utilizes a tet-off system, namely, transgene expression is shut-off in the presence of doxycycline. Indeed, supplementation of doxycycline (0.2 mg/ml) resulted in complete loss of HA-JDP2 expression (Figure 1E).

**Figure 2:**
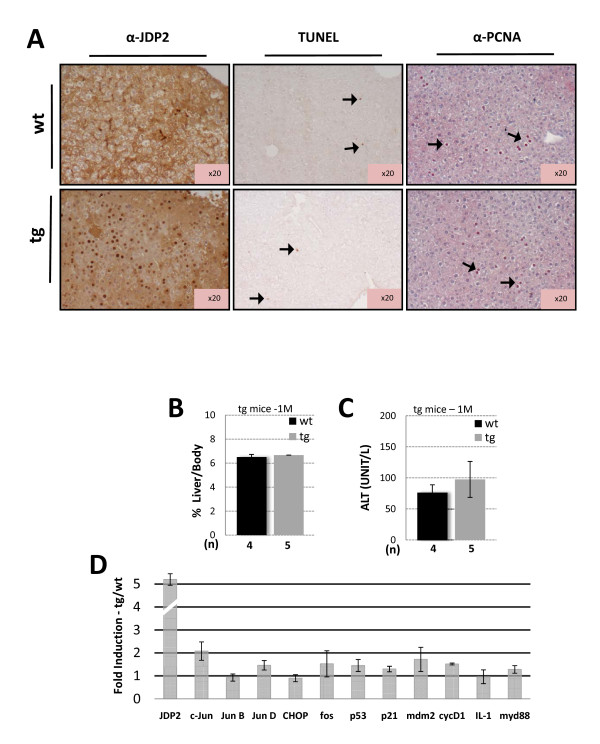
**JDP2 expression does not alter liver pathophysiology**. **A**. Immunohistochemistry of liver sections derived from either wild type (wt) or JDP2-transgenic (tg) mice. The expression of HA-JDP2 transgene was analyzed with anti-JDP2 antibody (left panel). The extent of apoptotic and proliferation of hepatocytes was analysed by TUNEL assay (middle panel) and PCNA staining (right panel). Arrows indicate positively stained cells. Liver/body weight ratio **(B.) **and Serum ALT **(C.) **was determined from one month old of either wild type control (wt, black bar) or JDP2-transgenic (tg, grey bar) mice. The results represent the average and SEM of the indicated number of animals (n). **D**. Real time qPCR of the indicated selected genes was performed from cDNA derived from mRNA extracted from liver of wild type and JDP2-transgenic mice. The results represent the calculated expression ratio between JDP-transgenic divided by the expression of the corresponding gene of the wild type mice. The average and SEM of three different experiments performed with pooled mRNA from at least three mice is presented.

We first examined the effect of JDP2 expression on liver pathophysiology. JDP2 is highly expressed in the nuclei of JDP2-transgenic hepatocytes (Figure 2A, left panel). The liver/body weight ratio is similar between JDP2-transgenic and wild type animals (Figure 2B). Similarly, serum alanine aminotransferase (ALT), a marker for hepatocyte damage, is not significantly altered between the genotypes (Figure 2C). We also measured the extent of apoptotic cells and hepatocyte proliferation by TUNEL and PCNA staining of liver sections and revealed no significant difference between the genotypes (Figure 2A). To reveal the expression level of several bZIP proteins and cell cycle regulators, we used real time PCR (qPCR) with cDNA derived from mRNA isolated from one month old mice. This analysis revealed that most of the genes tested were not significantly altered in cDNA derived from the JDP2-transgenic as compared with their wild type control (Figure 2D). c-Jun is the only gene whose transcription is modestly up-regulated. This was not expected, since c-Jun was previously described to be downregulated by JDP2 [19]. Collectively, mice with JDP2 over-expression in hepatocytes display no significant change in liver function.

**Figure 3:**
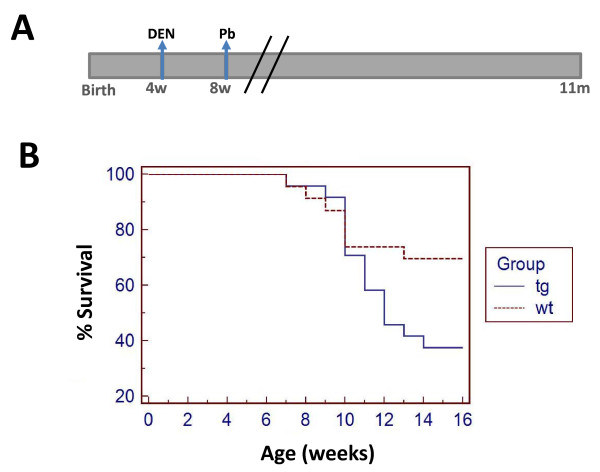
**Survival curve for the four weeks DEN protocol**. **A**. Schematic diagram is depicted for the four weeks DEN protocol. Mice were injected at four weeks of age with a single DEN injection (100 mg/Kg). At eight weeks of age the tumor promoter, 0.07% phenobarbital was provided in the drinking water in 5% sucrose. **B**. Mice survival was followed until eleven months of age. The survival of wild type (n = 23, dotted line) versus JDP2-transgenic (n = 24, solid line). Kaplan-Meier analysis performed shows P value < 0.05.

**Figure 4:**
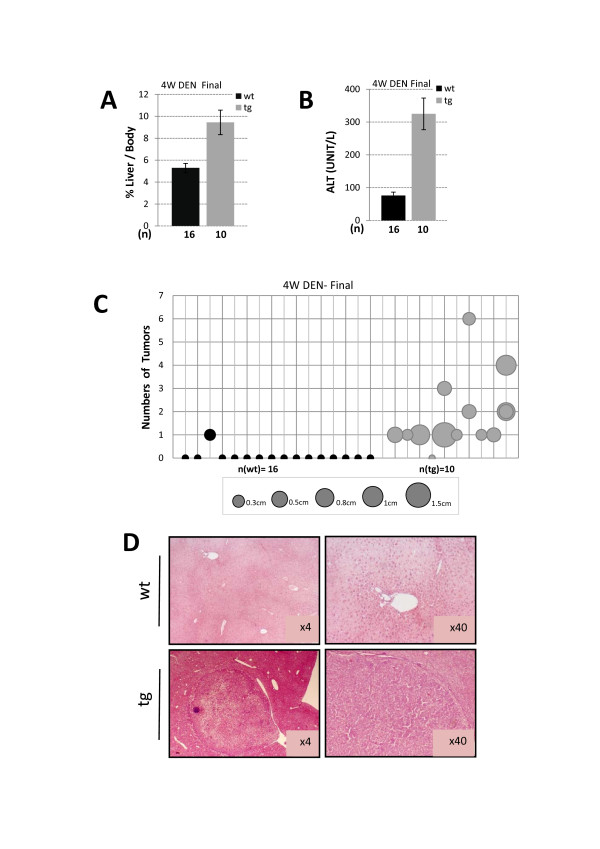
**Liver pathology of eleven months old DEN injected mice**. Liver/body weight ratio **(A.) **and Serum ALT **(B.) **was determined from eleven months old of either wild type control (wt) or JDP2-transgenic (tg) mice. The results represent the average and SEM of the indicated number of animals (n). **C**. The number of macroscopic tumors (Y axis) and their corresponding size are drawn for each mouse (X axis). The circle size corresponds to the tumor diameter according to the centimeter scale provided at the bottom of the figure. Student's t-test P value < 0.01 **D**. H&E staining of representative liver sections of DEN injected eleven months old either wild type control (wt) or JDP2-transgenic (tg).

To reveal the role of JDP2 expression in hepatocytes in a liver cancer model, we used a well established chemical carcinogen induced liver cancer protocol. In this model the tumors are initiated by the genotoxic drug diethylnitrosamine (DEN, 100 mg/Kg) at four weeks of age followed by tumor promoter Phenobarbital since eight weeks of age (Figure 3A). We first followed mice survival (Figure 3B). Whereas in wild type control mice 70% of the mice survived, within the first sixteen weeks of age, in JDP2-transgenic only 40% of the mice survived. Beyond four months of age, no further deaths were recorded. Dead mice did not display any sign of liver cancer. The precise cause of death was not determined. Surviving mice were sacrificed at seven months of age and their liver was analyzed. Wild type mice displayed 5.3% of liver/body weight, whereas, liver derived from JDP2-transgenic mice had a relatively enlarged liver corresponding to 9.5% liver/body weight (Figure 4A). Serum ALT levels were significantly higher in JDP2-transgenic mice (ALT-300 u/l) compared with wild type mice which had similar ALT levels as compared to one month old non-injected mice (Figure 4B). Nine out of ten JDP2-transgenic mice that survived beyond four months of age displayed numerous (between 1-8) visible macroscopic tumors (diameter between 0.3-1.5 cm) while only one out of sixteen liver derived from control wild type mice displayed one macroscopic tumor (Figure 4C). All other fifteen wild type mice showed no visible tumors at seven months of age. H&E stained liver sections demonstrated the existence of large tumors in JDP2-transgenic mice while in the control wild type mice only small nodules were readily observed (Figure 4D). Collectively, using the four weeks DEN liver cancer model, the JDP2-transgenic mice displayed a significantly lower survival rate and increased severity of liver cancer disease.

**Figure 5:**
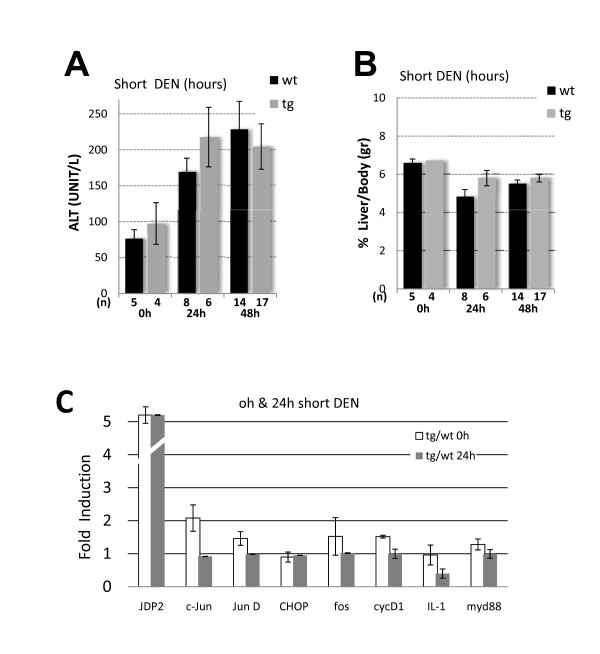
**JDP2 role in tumor initiation following DEN injection**. Mice were either not treated (0 h) or injected with DEN 100 mg/Kg and sacrificed 24 h and 48 h following injection. Serum ALT **(A.) **and Liver/body weight ratio **(B.) **were determined in wild type (wt, black bar) and JDP2-transgenic (tg, grey bar). The results represent the average and SEM of the indicated number of animals (n). **C**. The expression level of the indicated genes was determined by Real time qPCR to the indicated selected genes from cDNA derived from mRNA prepared from liver tissue of either wild type control (wt) or JDP2-transgenic (tg). The expression level of each gene was normalized by the 18s ribosomal RNA and β2-microglobulin mRNA. The mRNA derived from either non-injected (taken from data set Figure 2D) or mice injected with DEN (100 mg/Kg) sacrificed 24 h thereafter. The results represent the calculated expression ratio between JDP-transgenic divided by the expression level of the corresponding gene in the wild type mice. The average and SEM of three different experiments performed with pooled mRNA from the 0 h-n [tg = 4/wt = 5] and 24 h-n [tg = 3/wt = 2] animals.

**Figure 6:**
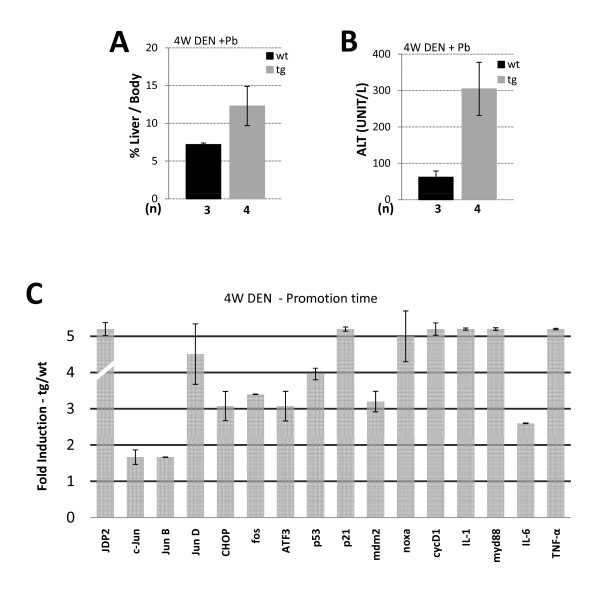
**Gene expression profile of DEN injected mice at four months of age**. Liver/body weight ratio **(A.) **and serum ALT **(B.) **were determined in wild type mice (wt, black bar) and JDP2-transgenic mice (tg, grey bar). The results represent the average and SEM of the indicated number of animals (n). **C**. The expression level of the indicated genes was determined by Real time qPCR to the indicated selected genes from cDNA derived from mRNA prepared from liver tissue of either wild type control (wt) or JDP2-transgenic (tg). The expression level of each gene was normalized by the 18s ribosomal RNA and β2-microglobulin mRNA. The mRNA derived from mice injected with DEN at four weeks of age followed by Phenobarbital treatment from eight weeks of age sacrificed at four months of age. The results represent the calculated expression ratio between JDP2-transgenic (tg) divided by the expression level of the corresponding gene in the wild type mice (wt). The average and SEM of three different experiments performed with pooled mRNA from n [tg = 4/w = 3] animals.

One possible explanation for the increase in disease severity in JDP2-transgenic mice might be that JDP2 expression potentiates tumor initiation. To examine JDP2's role in tumor initiation, mice were injected by a single DEN injection (100 mg/Kg) at four weeks of age. Mice were sacrificed 24 h and 48 h following injection and liver damage was assessed. Indeed, following DEN injection liver damage was increased by twofold as observed by the ALT level in both wild type and JDP2-transgenic mice but with no significant difference between the two genotypes (Figure 5A). In addition, liver to body weight was not significantly altered in both genotypes (Figure 5B). Gene expression profile of numerous selected genes was un-affected 24 h following DEN injection between wild type and JDP2-transgenic (Figure 5C). To examine the changes that occur during liver promotion stages, mice were injected with DEN at four weeks of age treated with Phenobarbital at eight weeks of age. Mice were sacrificed at four months of age. Liver/body weight ratio was twofold higher in JDP2-transgenic mice compared with age matched DEN injected wild type control mice (Figure 6A). In addition, serum ALT levels were six fold higher in JDP2-transgenic mice already at four months of age (Figure 6B). We analyzed the pattern of gene expression of selected genes by qPCR. Surprisingly, JDP2-transgenic mice displayed potentiation of expression of numerous genes including: ATF3, CHOP10, cFos and JunD (Figure 6C). In addition, inflammatory cytokines such as IL-1, IL-6 and TNFα were highly elevated. Moreover, p53 mRNA level and its target genes p21 and Mdm2 transcription level were significantly increased in the liver of JDP2-transgenic mice (Figure 6C). c-Jun expression was not altered as compared with one month old non-injected mice. Collectively, the data suggests that a dramatic increase in the expression of key regulatory proteins occurs in JDP2-transgenic mice during the tumor promotion stage.

**Figure 7:**
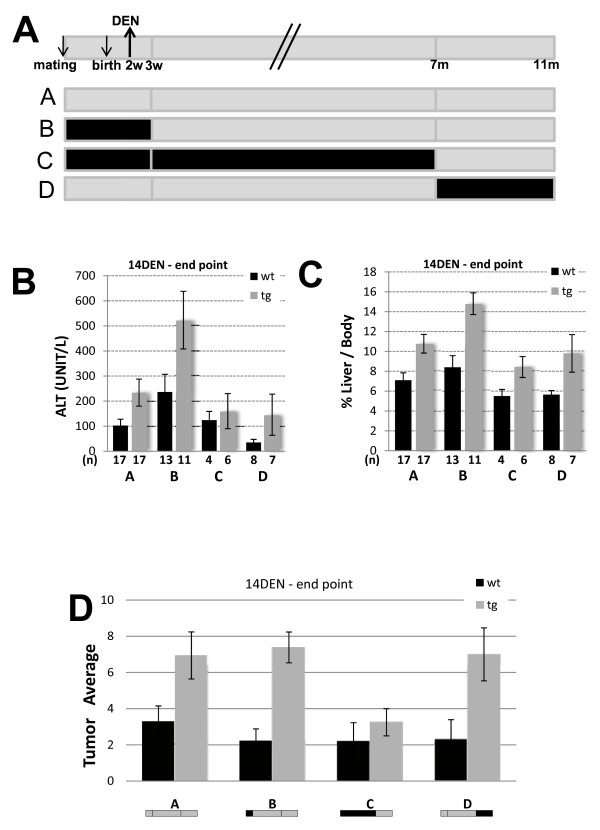
**JDP2-tg and liver cancer in 14 days DEN protocol**. Mice were injected with DEN 25 mg/Kg at 14 days postnatal and sacrificed at eleven months of age. **A**. Schematic diagram representing the 14 day DEN protocol and doxycycline treatments used in the indicated experimental groups. Mice were treated with doxycycline 0.2 mg/ml dissolved in 5% sucrose in the drinking water as indicated in the scheme (black line, no JDP2 expression). Grey line represents JDP2 expression. Serum ALT **(B.) **and Liver/body weight ratio **(C.) **was determined. The results represent the average and SEM of the indicated number of animals (n). **D**. The number of macroscopic tumors in liver derived from eleven months old mice injected with DEN and treated as depicted schematically for each experimental group. Wild type (wt, black bar) and JDP2-transgenic (tg). The results represent the average and SEM of the number (n) of animals in each group. Asterisks represent P values < 0.05

To assess the role of JDP2 in a different DEN induced liver cancer protocol, we used a second well established protocol in which a single injection of DEN (25 mg/Kg) at 14 days postnatal [8]. Unlike the four weeks DEN protocol, mice treatment at the earlier age does not require the assistance of tumor promoter to develop liver cancer. Using this model 100% of injected mice develop macroscopic liver tumors at eleven months of age [8]. To identify the importance of the time of JDP2 expression at the different stages of the development of liver cancer, we took advantages of the ability to shut-off the transgene expression using doxycycline. We studied three groups of mice in which JDP2 expression was shut-off during different stages of liver cancer development: initiation (Figure 7A, group B), initiation + promotion (Figure 7A, group C) and progression (Figure 7A, group D). Wild type control mice receiving identical doxycycline treatment were included in each of the three experimental cohorts.

**Figure 8:**
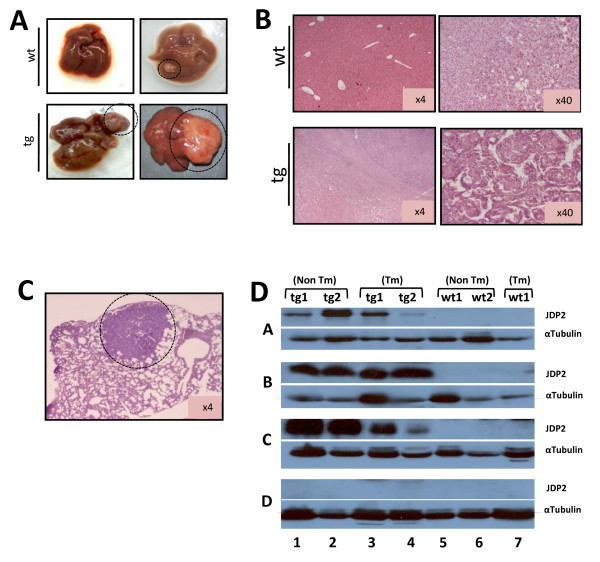
**Liver tumors analysis for 14 days DEN protocol**. **A**. Representative liver photos derived from either wild type control (wt) and JDP2-transgenic (tg) mice are shown. **B**. Liver sections stained with H&E of either wild type or JDP2-tg. **C**. Lung section with metastatic liver tumor derived from JDP2-tg mice is shown. **D**. Western blot analysis of lysate derived from eleven months old mice of either wild type (wt 1-2, lanes 5-7) or JDP2-transgenic (tg 1-2, lanes 1-4) mice. Lysate was prepared from liver (non Tm, lanes 1-2 and 5-6) and liver tumors (Tm, lanes 3-4 and 7) from the same mouse cohort (indicated by a number) of the doxycycline treated mice as shown in A. Tumors were extracted only from wild type mice #1 representing the different experimental groups. Membranes were probed with anti-JDP2 (top lane in each experimental group) and anti-α-tubulin (bottom lane in each experimental group).

Consistent with the four weeks DEN protocol, mice expressing JDP2 in the liver displayed increased disease severity. Both serum ALT levels and liver/body weight ratio were significantly higher in JDP2-transgenic mice as compared with wild type mice (Figure 7B and 7C; group A). The number of macroscopic tumors was doubled in JDP2-transgenic mice as compared with the wild type mice (Figure 7D; group A). In addition, the tumors derived from the JDP2-transgenic mice were bigger as compared with wild type control mice. Representative macroscopic and microscopic tumors are shown (Figure 8A and 8B). In addition, some of the JDP2-transgenic mice displayed lung metastases (Figure 8C). Collectively, using the 14 day DEN protocol, JDP2-transgenic resulted in an increase in the severity of liver cancer. Similar results were obtained with mice that were either treated with doxycycline during pregnancy through three weeks postnatal (Group B) or mice that were treated with doxycycline from seven months of age (group D). Interestingly, JDP2-trangenic mice treated with doxycycline from pregnancy till seven months (Group C) displayed similar ALT level and comparable number of tumors to wild type control mice (Figure 7D). Western blot analysis of liver lysates derived from all four experimental groups revealed that doxycycline treatment resulted in complete loss of transgene expression (Figure 8D group D). Moreover, JDP2 expression was observed in the liver and tumors derived from JDP2-transgenic mice. This result suggesting, that liver tumors observed in JDP2-transgenic do not represent hepatocyte that lost JDP2 expression. Whereas, lack of expression of JDP2 either at the initiation stage or the progression stages had no effect on the disease severity, the lack of JDP2 expression during the initiation and promotion stage significantly reduced the number of tumors and severity of the disease. These results suggest that the expression of JDP2 during the promotion stage plays a critical role in the increased severity of liver cancer in this model.

## Discussion

The role of JDP2 in carcinogenesis is a matter of debate. On the one hand, JDP2 over-expression results in cell cycle arrest [20] and reduced foci formation in Ras dependent cell transformation [23]. In addition, prostate cancer cell lines with JDP2 over-expression form smaller tumors in mice [23]. On the other hand, JDP2 over-expression in chicken embryo fibroblasts imparts a partial oncogenic phenotype [30]. Moreover, JDP2 locus was identified at viral integration sites resulting in T cell lymphoma [24-26,31]. Most of JDP2 proteins that are expressed as a result of the viral integration lack the N-terminal JDP2 domain, yet some integrations enhance the expression of full length JDP2 [25,31]. The mechanisms through which JDP2 acts as an oncogene remain to be determined. In addition, whether or not JDP2 plays a role in carcinogenesis in humans is yet to be demonstrated.

Here we described the generation of JDP2-transgenic mice that express JDP2 specifically in the liver. Although JDP2-expressing mice highly express JDP2, JDP2-transgenic mice display no liver dysfunction phenotype unless faced with specific perturbations such as DEN administration. Thus, c-Jun inhibition by JDP2 during pregnancy is effective only from day 10-12 and therefore may bypass the critical timing resulting in the adverse effect of inhibition of fetal liver development. Similar results were obtained with dominant negative IkB transgenic mice that were able to complete full term and still efficiently inhibit NFkB activity in adult mice [32]. It appears that the LAP promoter that is driving the expression of the tTA is expressed at extremely low levels in utero. Therefore, the transgene expression is insufficient to completely inactivate either NFkB or c-Jun both of which are required for liver development.

c-Jun was found to promote liver cancer. Mice with c-Jun conditional knockout in the liver display reduced liver cancer development following the well established DEN model. c-Jun-KO mice displayed increased apoptosis in the liver. c-Jun was suggested to act through a p53 dependent mechanism and activation of the pro-apoptotic gene Noxa [16]. Previous studies directed towards inhibition of c-Jun transcription activity used c-Jun lacking its transcription activation domain, TAM67. Indeed, expression of TAM67 in the skin resulted in inhibition of tumor promotion in two stage skin carcinogenesis model [33]. JDP2 was previously shown to inhibit cell proliferation *in vitro *and directly inhibit the transcription of cell cycle proteins such as cyclin D1 [20]. In view of these results, we expected that JDP2 over-expression in the liver will mimic c-Jun disruption and thus may result in inhibition of liver cancer development in this model. In contrast, JDP2 potentiated the severity of liver cancer in the two DEN induced hepatocellular cancer models. JDP2-transgenic mice displayed numerous enlarged visible tumors and higher liver/body ratio as well as increased level of liver damage. Gene expression analysis revealed an increase in the inflammatory response at four months old JDP2-transgenic mice. Interestingly, CHOP10 is expressed at high levels in JDP2-transgenic mice. We have demonstrated that JDP2 can act as a strong transcription activator depending on the leucine zipper protein member it is associated with. CHOP10 protein can form stable heterodimer with JDP2 and is found to activate transcription from TRE dependent genes [34]. Indeed, CHOP10 expression level is highly induced in mice injected with DEN during the promotion stage as well. The mechanism by which CHOP10 expression is augmented is yet to be determined. The potentiation of TRE dependent genes such as those elevated during the promotion stage in JDP2-trangenic mice could be the result of JDP2-CHOP10 transcription activity. In addition to CHOP10, ATF3 expression level is highly elevated in the liver of four months old JDP2-transgenic mice. Interestingly, both CHOP10 and ATF3 were identified as JDP2 target genes [35,36]. ATF3 was found to act as an oncogene in breast tumors [37]. Therefore, ATF3 high expression levels observed in the liver of JDP2-transgenic mice may a play a positive role at the tumor promotion stage of liver cancer.

The mechanism by which the impaired HCC development following c-Jun disruption was proposed to be due increased hepatocyte apoptosis in a cell autonomous manner [16]. In contrast, using the DEN model in mice with liver disruption of IKKβ, it appears that hepatocyte death is the main driving force that promotes cell proliferation leading to liver cancer development in a non cell autonomous manner [8]. Retroviral activation of JDP2 in T-cell lymphomas of mice is the only evidence for a gain-of-function potential of JDP2 in cancer development. Although the fact that most of the resulted JDP2 transcripts lacks the N-terminal histone acetylation inhibitory (INHAT) domain [38], some tumors represent elevation of canonical full length JDP2 transcript [31]. Expression of low levels of JDP2 together with mutant NRas (G12D) resulted in anchorage independent growth [31]. In contrast, JDP2 over-expression resulted in inhibition of Ha-Ras dependent foci formation in NIH3T3 [23]. It was postulated that high JDP2 expression level results in a cellular toxicity and therefore, inhibited cellular transformation by Ras. However, in the liver such cellular toxicity may serve as the driving force in potentiating liver cancer through compensatory proliferation mechanism [10]. Collectively, it seems that JDP2 plays a protective role in a cell autonomous manner *in vitro *but the same process can have a positive outcome in a non cell autonomous way resulting in potentiation of liver cancer.

To examine whether or not JDP2 has an effect at the tumor initiation stage, we tested the expression of genes 24 h following DEN injection. The expression of numerous genes revealed no significant alteration of expression between wild type and JDP2 transgenic mice. This suggested that liver damage and tumor initiation is alike in mice independent of JDP2 expression. Consistently, JDP2-transgenic mice in which transgene expression was suppressed during the first three weeks of life displayed very similar liver cancer severity as compared with a mice cohort in which JDP2 was expressed at all stages. In contrast, mice in which JDP2 expression was suppressed during the initiation and promotion stages showed similar liver cancer levels as compared with wild type mice. Collectively, JDP2 expression during the promotion stage is found to be important for potentiation of liver cancer by acting as a tumor promoter in the DEN model.

## Conclusions

JDP2 over-expression potentiaes DEN induced liver cancer development. JDP2 role at the promotion stage is demonstrated. The increase of CHOP10 expression may provide a possible explanation for JDP2 potentiation of gene transcription at the promotion phase of liver cancer development. Collectively, JDP2 plays a dual role in carcinogenesis depending on the cellular and tissue context.

## Methods

### Transgenic Mice

All studies involving mice were performed according to the protocol approved by the Technion Animal Inspection Committee. The Technion holds an NIH animal approval license, number A5026-01.

### Mice strain

#### LAP mice

Mouse strain with tetracycline activator (tTA) liver expression under the control of a tissue-specific C/EBPβ (LAP) promoter [28]. The system represents the Tet-off system. Doxycycline hydrochloride (D-9891, Sigma-Aldrich) 0.2 mg/ml is dissolved in 5% sucrose and supplied in the drinking water to counteract the bitter taste of the antibiotic.

#### JDP2 transgenic mice

The tet-promoter is designed to drive bi-directionally the expression of β-galactosidase gene and HA-JDP2 [27]. Crossing the homozygous LAP-driver with the heterozygous JDP2-responder, single copies of the tTA and JDP2 are present in the double transgenic mice (JDP2-transgenic, tg), and a single copy of the tTA transgene (control, wt).

#### Mouse Genotyping

Mouse genotyping is routinely performed by PCR on genomic DNA extracted from mouse tail (XNAT, Sigma-Aldrich). PCR Primers used for genotyping: JDP2F atgatgcctgggcagatccca, JDP2R tcacttcttgtccagctgctcc, tTAF gctgcttaatgaggtcgcaatcg, tTAR gccccacagcgctgagtgcat. PCR was performed using RedyMix™ reaction mix (R2648, Sigma-Aldrich)

#### DEN protocol

Mice were injected intraperitoneally with DEN either with 25 mg/Kg at two weeks of age or 100 mg/Kg at four weeks of age followed by addition of 0.07% phenobarbital in the drinking water containing 5% sucrose at eight weeks of age.

#### Blood analysis

Blood samples were rapidly taken from the heart of anesthetized mice. Serum was separated and alanine aminotransferase (ALT) level was determined at the Biochemistry Department Rambam Medical Center.

#### Harvesting mouse liver

Livers were excised from anesthetized animals. Externally visible nodules and tumors were counted and measured. Part of the liver was fixed in 4% buffered formaldehyde and paraffin embedded for histological analysis, and the remaining liver tissue was quickly frozen in liquid nitrogen and stored at -80°c until use.

### Immunohistochemical Analysis

Tissue sections (6 μm) were stained with Hematoxylin and Eosin (H&E) for general morphology or with antibodies against JDP2 [17] and PCNA (Santa-Cruz, SC-7907). Immunostaining was performed using the EnVision™ G\2 System alkaline phosphatase kit (DakoCytomation K5355, Dako). Staining was performed according to the manufacturers' instructions. Sections were visualized using an Olympus light microscope.

**Apoptosis **was determined by the TUNEL staining kit. Sections were stained by the *in situ *death detection POD kit (Roche Diagnostics). **Proliferation **was determined by anti-PCNA staining (Santa-Cruz, SC-7907).

### Statistical Analysis

Data are expressed as means ± SEM in n number of experiments. Differences were analyzed by Student's t test. P values < 0.05 were considered significant. Overall survival analysis was studied by Kaplan-Meier curves.

### Nuclear Extract and Western blotting

Western blotting with liver nuclear protein extract was performed as previously described [34].

JDP2 [17] and anti-HA antibodies were used at 1:500 dilution, anti-α-tubulin (Sigma-Aldrich) was used at 1:5000 dilution.

### mRNA analysis

RNA was extracted from 30 mg liver tissue using RNeasy Kit according to the manufacturers' instructions (Qiagen Inc.). Single-stranded cDNA were synthesized by reverse transcription using Verso™ cDNA Kit (Thermo Scientific AB-1453/A) with random hexamer primer. Prior to cDNA preparation samples were incubated at 70°C for 5 minutes. Real-time qPCR was performed with the Rotor-Gene 6000TM (Corbett) using absolute blue SYBER green ROX mix (Thermo Scientific AB-4162/B). Values were normalized with 18s and β2-microglobulin levels for each sample. Primer sequences are provided:

Sequence - Primer Name

TNF-α- F-ccagaccctcacactcagatca R-cacttggtggtttgctacgac

18s - F-tagagggacaagtggcgttca R-cccggacatctaagggcat

Jun-D - F-ggcgggattgaaaccaggg R-agcccgttggactggatga

IL-1 - F-gcaccttacacctaccagagt R-aaacttctgcctgacgagctt

Myd88 - F-aggacaaacgccggaactttt R-gccgatagtctgtctgttctagt

p21 - F-cgagaacggtggaactttgac R-cagggctcaggtagaccttg

Mdm2 - F-aggagatgtgtttggagtccc R-ctcagcgatgtgccagagtc

Noxa - F-gcagagctaccacctgagttc R-cttttgcgacttcccaggca

p53 - F-ctctcccccgcaaaagaaaa R-ctcctctgtagcatgggcatc

CHOP10 - F-gggccaacagaggtcacac R-cttcatgcgttgcttccca

IL-6 - F-tagtccttcctaccccaatttcc R-ttggtccttagccactccttc

c-Jun - F-ccttctacgacgatgccctc R-ggttcaaggtcatgctctgttt

JUNB - F-tcacgacgactcttacgcag Rccttgagaccccgataggga

JDP2 - F-ctcactcttcacgggttgg R-gctgaaatacgctgacatc

fos - F-ccagcagaagttccgggtag R-gtagggatgtgagcgtggata

CycD1 - F-gcgtaccctgacaccaatctc R-ctcctcttcgcacttctgctc

ATF3 - F-gaggattttgctaacctgacc R-ttgacggtaactgactccc

β2-microglubulin - F-ttctggtgcttgtctcactga R-cagtatgttcggcttcccattc

## List of abbreviation

ALT: Alanine aminotransferase; AP-1: Activating protein 1; ATF3: Activating transcription factor 3; bZIP: basic leucine zipper; CHOP10: C/EBP homologous protein 10; DEN: diethylnitrosamine; HCC: Hepatocellular carcinoma; IkB: Inhibitor of kB; IKKβ: IkB kinase β; JDP2: c-Jun dimerization protein 2; LAP: liver activating protein; NFkB: Nuclear factor kB; tTA: tetracycline transactivator.

## Competing interests

The authors declare that they have no competing interests.

## Authors' contributions

KBW carried out all molecular biology and animal experiments EP participated in study design and performed all pathological analysis and helped draft the manuscript AA carried out all animal experiments, designed and drafted the manuscript. All authors read and approved the final manuscript.
